# Acid-suppressive medications during pregnancy and risk of asthma and allergy in the offspring: protocol for a systematic review

**DOI:** 10.1038/npjpcrm.2016.1

**Published:** 2016-03-03

**Authors:** Rebecca E Devine, Aziz Sheikh, Bright I Nwaru

**Affiliations:** 1 Asthma UK Centre for Applied Research, Centre for Medical Informatics, Usher Institute of Population Health Sciences and Informatics, The University of Edinburgh, Edinburgh, UK; 2 School of Health Sciences, University of Tampere, Tampere, Finland

## Background

Asthma and allergy are leading causes of morbidity in childhood, and in some cases may lead to mortality.^1^ Although the prevalence of asthma may have reached a plateau in some developed country settings, data suggest that the prevalence of other allergic disorders, such as food allergy, may be rising.^2^ The overall burden of asthma and allergy transcends individual, familial and societal contexts, and it presents a challenge to public healthcare systems in terms of prevention and management of symptoms.^3–8^ The propensity to develop asthma or allergy is at least partially established before birth, resulting from the interplay between genetic predisposition and environmental exposures.^9,10^

It is now increasingly understood that known and unknown prenatal environmental exposures play their part, importantly during fetal development.^11^ A number of studies have investigated the role of several fetal environmental exposures, including maternal obesity, inhaled pollutants (such as tobacco smoke and diesel exhaust fumes) and ingestion of paracetamol during pregnancy.^12–16^ In addition to these prenatal exposures, recent emerging evidence indicates that the use of acid-suppressive medications by mothers during pregnancy may increase the risk of development of asthma and allergy in the offspring.^17–20^ Reported increased risk of asthma when the expectant mother is exposed to acid-suppressive drugs ranges from 32%^19^ to 57%,^21^ although for other allergic diseases reported data are inconsistent.

The precise underlying pathophysiological mechanism for the role of acid-suppressive medications in the development of asthma is not clearly understood. Dehlink *et al.* ^17^ suggested three mechanisms by which maternal acid-suppressive therapy may promote allergy in the progeny: (i) gastric acid suppression could interfere with digestion of labile antigens in the maternal stomach and increase the amount of allergen the foetus is exposed to and results in sensitisation; (ii) acid-suppressive medications may induce a Th2 cytokine pattern in mothers, promoting an allergy-prone state in the fetus; or (iii) maternal allergen-specific immunoglobulin E could cross fetal membranes and induce sensitisation of fetal immune cells to food and airborne allergens before birth. In a study conducted in adults, immunoglobulin E sensitisation was detected five months after discontinuation of treatment with acid-suppressive medication.^22^

Acid-suppressive medications are commonly used in the management of gastro-oesophageal reflux and heartburn symptoms, which are frequently reported by expectant mothers.^23,24^ Although the prescription of any drug should be carefully re-evaluated during pregnancy, some substances have become over-the-counter drugs for pregnant women even if their precise impact on the fetus has not been definitively evaluated.^25^ Given the widespread use of gastric-acid-suppressing medications during pregnancy, their role in the development of asthma and allergic disorders in the offspring raises potential public health concern. To appreciate the emerging evidence base, there is now a need to comprehensively synthesise the available primary studies. By bringing together all relevant data, the underlying role of acid-suppressive medications in the development of asthma and allergy could be clarified, which would eventually provide the opportunity for initiating primary prevention interventions.

## Aims and objectives

We aim to identify, critically appraise and undertake meta-analysis of studies on the association between the use of acid-suppressive medications during pregnancy and the development of asthma and allergic disorders in the offspring.

Specific objectives are as follows:

1. To study the associations between maternal use of H2 receptor antagonists, proton pump inhibitors and antacids during pregnancy and the risk of asthma and allergy in the offspring.
2. To identify whether the association between acid-suppressive medications (H2 receptor antagonists, proton pump inhibitors and antacids) and asthma and allergy varies according to the (i) timing; (ii) frequency; or (iii) dose of intake.

## Methods

### Study eligibility criteria

#### Types of studies

We will include all cohort studies, case–control studies and cross-sectional studies investigating the association between acid-suppressive medications in pregnant mothers and allergy/asthma outcomes in their offspring. We will exclude reviews, case studies and case series, and animal studies. We will include conference abstracts in a table of unpublished studies. There will be no minimum number of participants or minimum length of follow-up required in the studies to be considered for inclusion.

#### Types of participants

We will include studies involving women of any age during preconception and pregnancy and their offspring who are <17 years old.

#### Types of exposure

We will include all studies that have investigated the associations between maternal use of acid-suppressive medications (H2 receptor antagonists, proton pump inhibitors and antacids) during pregnancy and the risk of asthma and allergy in the offspring.

#### Types of outcome measures

Primary outcomes will include self-reported or objectively defined asthma, atopic dermatitis/eczema, allergic rhinitis or hayfever; anaphylaxis and urticaria and food allergy reported by physician or hospital record; and atopic sensitisation as defined either by skin-prick test or raised antigen-specific immunoglobulin E.

Secondary outcomes will include objective and subjective measures of disease severity and impact on quality of life, including asthma exacerbations, the use of asthma medications, hospitalisation for asthma, wheeze as defined by self-report or objective diagnosis, indicators of airway function (peak expiratory flow, forced expiratory volume in 1 s, forced vital capacity, forced expiratory flow rate or alternative age appropriate pulmonary function tests (oscillometry or exhaled nitric oxide analysis)) and measures of health-related quality of life.

### Search strategy

To identify studies for the review, we will search the following international electronic databases: MEDLINE (Ovid), EMBASE (Ovid), Web of Science CORE (Thomson Reuters), BIOSIS (Thomson Reuters), CINAHL (Cumulative Index to Nursing and Allied Health Literature; EBSCO), Cochrane Library (CDSR; Wiley), Global Health CABI (Ovid), Global Health Library (Global Index Medicus), Scopus, Popline and Google Scholar. Additional studies will be retrieved by searching the references of identified eligible papers and by contacting a panel of international experts on the topic. Conference abstracts will be retrieved through search of ISI Conference Proceedings Citation Index via Web of Knowledge and ZETOC (British Library). Unpublished and in-progress studies will be identified by searching online trial registries such as Current Controlled Trials, ClinicalTrials.gov, Australian and New Zealand Clinical Trials Registry. All databases will be searched from inception to 2015. No language restrictions will be imposed; for foreign language publications, translations will be sought where possible. All eligible studies identified will be manually checked to trace any original studies not identified via this search. Supplementary Appendix 1 presents details of our search strategy, which was developed in MEDLINE and will be adapted in searching other databases.

#### Screening of retrieved literature

The titles and abstracts of all papers retrieved from the databases will be checked independently by two reviewers against the criteria of the study. The full texts of papers that are potentially eligible will be retrieved and further assessed for inclusion independently by two reviewers. Any discrepancies in the screening processes between the two reviewers will be resolved by consensus, and disagreements will be arbitrated by a third reviewer.

### Data extraction

A customised data collection form will be used by two reviewers, independently, to extract relevant study data from full-text papers selected for inclusion. The form will be piloted and refined before being applied to all full-text reports. Where necessary, clarification and additional data will be sought from study authors. Key findings from each included study will be summarised and tabulated. Any discrepancies in data extraction between the two reviewers will be resolved by consensus, and disagreements will be arbitrated by a third reviewer.

### Quality assessment

Quality assessment and risk of bias assessment of studies will be undertaken using the Effective Public Health Practice Project tool (http://www.ephpp.ca/).

### Data synthesis

Data will be presented in a narrative and tabular form. Where possible, meta-analysis will be performed on clinically, methodologically and statistically comparable studies (comparable particularly with respect to study design, exposure measures and assessment, and outcomes and assessment) using random-effects models. Heterogeneity will be assessed using the I-squared statistic. Where possible, depending on reporting in original studies, sensitivity and subgroup analyses will be performed. Subgroup analysis will be performed based on participants’ characteristics, including the trimester at the time of intake of acid-suppressive medications, dosage and duration of intake. Sensitivity analysis will be performed to explore the source of heterogeneity, e.g., based on study quality or risk of bias. Publication bias will be explored using funnel plots and will be estimated using Begg and Egger tests.^26,27^ Comprehensive Meta-Anaysis software will be used for the meta-analysis.

### Registration and reporting

This study will be registered on the University of York Centre for Reviews and Dissemination International prospective register of systematic reviews (PROSPERO). We will report according to the PRISMA guidelines for reporting of systematic reviews and MOOSE guidelines for observational epidemiological systematic reviews.^28,29^

## Discussion and conclusion

Current understanding indicates that the fetal period is critical for the onset of common chronic disorders later in childhood, including asthma and allergy, whose susceptibility is now believed to be already established *in utero* as a result of the interplay of both genetic and environmental factors on the developing immune system.^11,30^ The identification of specific modifiable environmental factors offers a real possibility of enhancing our understanding of the underlying disease mechanisms and development of early potential preventive strategies.

The emerging evidence indicating that prenatal use of acid-suppressive medications may increase the risk of asthma and allergy in the offspring may constitute a public health concern given that these medications are commonly used for the treatment of gastro-oesophageal reflux and heartburn symptoms during pregnancy. However, given mixed evidence now reported by studies on this topic, a comprehensive synthesis of the evidence is essential in the following: (i) clarifying the putative role of acid-suppressive medications in the development of asthma and allergy in children; (ii) clarifying whether the different commonly used acid-suppressive medications (H2 receptor antagonists, proton pump inhibitors and antacids) and their doses are differentially associated with the risk of asthma and allergy in children; (iii) clarifying whether there is a critical time window of exposure to acid-suppressive medications during pregnancy that maximises the risk of asthma and allergy in the offspring; (iv) identifying subgroups of mothers and their offspring that may benefit or are at high risk of exposure to prenatal exposure to acid-suppressive medications; and (v) identifying potential research gaps in this evidence base that will need to be taken into account going forward.

We anticipate that there may be clinical, methodological and statistical heterogeneity in the studies we identify for inclusion. These factors will be taken into consideration in the decision on whether to pool data and also to ensure appropriate interpretation of findings.

## Notes

AS is Joint Editor-in-Chief of *npj Primary Care Respiratory Medicine*, but was not involved in the editorial review of, nor the decision to publish, this article. The other authors declare no conflict of interest.

## References

[bib1] Masoli, M. , Fabian, D. , Holt, S. & Beasley, R. The global burden of asthma: executive summary of the gina dissemination committee report. Allergy 59, 469–478 (2004). 15080825 10.1111/j.1398-9995.2004.00526.x

[bib2] Asher, M. I. et al. Worldwide time trends in the prevalence of symptoms of asthma, allergic rhinoconjunctivitis, and eczema in childhood: Isaac phases one and three repeat multicountry cross-sectional surveys. Lancet 368, 733–743 (2006). 16935684 10.1016/S0140-6736(06)69283-0

[bib3] Ozdoganoglu, T. & Songu, M. The burden of allergic rhinitis and asthma. Ther. Adv. Respir Dis. 6, 11–23 (2012). 22179899 10.1177/1753465811431975

[bib4] Hofmaier, S. Allergic airway diseases in childhood: an update. Pediatr. Allergy Immunol. 25, 810–816 (2014). 25469999 10.1111/pai.12322

[bib5] Gupta, R. S. et al. The prevalence, severity, and distribution of childhood food allergy in the united states. Pediatrics 128, e9–e17 (2011). 21690110 10.1542/peds.2011-0204

[bib6] Meltzer, E. O. et al. Burden of allergic rhinitis: results from the pediatric allergies in america survey. J. Allergy Clin. Immunol. 124, S43–S70 (2009). 19592081 10.1016/j.jaci.2009.05.013

[bib7] Carroll, C. L. , Balkrishnan, R. , Feldman, S. R. , Fleischer, A. B. & Manuel, J. C. The burden of atopic dermatitis: Impact on the patient, family, and society. Pediatr. Dermatol. 22, 192–199 (2005). 15916563 10.1111/j.1525-1470.2005.22303.x

[bib8] O'connell, E. The burden of atopy and asthma in children. Allergy 59, 7–11 (2004). 15245350 10.1111/j.1398-9995.2004.00563.x

[bib9] Cole Johnson, C. et al. Environmental epidemiology of pediatric asthma and allergy. Epidemiol. Rev. 24, 154–175 (2002). 12762090 10.1093/epirev/mxf013

[bib10] Busse, W. W. & Lemanske, R. F. Asthma. N. Engl. J. Med. 344, 350–362 (2001). 11172168 10.1056/NEJM200102013440507

[bib11] Warner, J. The early life origins of asthma and related allergic disorders. Arch. Dis. Child. 89, 97–102 (2004). 14736614 10.1136/adc.2002.013029 PMC1719801

[bib12] Hatzler, L. , Hofmaier, S. & Papadopoulos, N. G. Allergic airway diseases in childhood–marching from epidemiology to novel concepts of prevention. Pediatr. Allergy Immunol. 23, 616–622 (2012). 23106446 10.1111/pai.12022

[bib13] Reichman, N. E. & Nepomnyaschy, L. Maternal pre-pregnancy obesity and diagnosis of asthma in offspring at age 3 years. Matern. Child Health J. 12, 725–733 (2008). 17987372 10.1007/s10995-007-0292-2

[bib14] Gilliland, F. D. , Li, Y.-F. & Peters, J. M. Effects of maternal smoking during pregnancy and environmental tobacco smoke on asthma and wheezing in children. Am. J. Respir. Crit. Care Med. 163, 429–436 (2001). 11179118 10.1164/ajrccm.163.2.2006009

[bib15] Fedulov, A. V. et al. Pulmonary exposure to particles during pregnancy causes increased neonatal asthma susceptibility. Am. J. Respir. Cell Mol. Biol. 38, 57–67 (2008). 17656681 10.1165/rcmb.2007-0124OC PMC2176127

[bib16] Shaheen, S. et al. Prenatal paracetamol exposure and risk of asthma and elevated immunoglobulin e in childhood. Clin. Exp. Allergy 35, 18–25 (2005). 15649261 10.1111/j.1365-2222.2005.02151.x

[bib17] Dehlink, E. , Yen, E. , Leichtner, A. , Hait, E. & Fiebiger, E. First evidence of a possible association between gastric acid suppression during pregnancy and childhood asthma: A population‐based register study. Clin. Exp. Allergy 39, 246–253 (2009). 19134022 10.1111/j.1365-2222.2008.03125.x

[bib18] Andersen, A. B. et al. Prenatal exposure to acid-suppressive drugs and the risk of childhood asthma: a population-based danish cohort study. Aliment. Pharmacol. Ther. 35, 1190–1198 (2012). 22443179 10.1111/j.1365-2036.2012.05073.x

[bib19] Kallen, B. , Finnstrom, O. , Nygren, K. G. & Otterblad Olausson, P. Maternal drug use during pregnancy and asthma risk among children. Pediatr. Allergy Immunol. 24, 28–32 (2013). 23331527 10.1111/pai.12034

[bib20] Mulder, B. et al. Acid-suppressive drug use in pregnancy and the toddler's asthma risk: A crossover, case-control study. J. Allergy Clin. Immunol. 132, 1438–1440 (2013). 23992747 10.1016/j.jaci.2013.07.012

[bib21] Mulder, B. et al. Prenatal exposure to acid‐suppressive drugs and the risk of allergic diseases in the offspring: a cohort study. Clin. Exp. Allergy 44, 261–269 (2014). 24164287 10.1111/cea.12227

[bib22] Untersmayr, E. et al. Anti-ulcer drugs promote ige formation toward dietary antigens in adult patients. FASEB J. 19, 656–658 (2005). 15671152 10.1096/fj.04-3170fje

[bib23] Ali, R. A. R. & Egan, L. J. Gastroesophageal reflux disease in pregnancy. Best Pract. Res. Clin. Gastroenterol. 21, 793–806 (2007). 17889808 10.1016/j.bpg.2007.05.006

[bib24] Olans, L. & Wolf, J. Gastroesophageal reflux in pregnancy. Gastrointest. Endosc. Clin. North Am. 4, 699–712 (1994). 7812642

[bib25] Servey, J. & Chang, J. Over-the-counter medications in pregnancy. Am. Fam. Physician 90, 548–555 (2014). 25369643

[bib26] Begg, C. B. & Mazumdar, M. Operating characteristics of a rank correlation test for publication bias. Biometrics 50, 1088–1101 (1994). 7786990

[bib27] Egger, M. , Smith, G. D. , Schneider, M. & Minder, C. Bias in meta-analysis detected by a simple, graphical test. Br. Med. J. 315, 629–634 (1997). 9310563 10.1136/bmj.315.7109.629 PMC2127453

[bib28] Moher, D. , Liberati, A. , Tetzlaff, J. & Altman, D. G. Preferred reporting items for systematic reviews and meta-analyses: the prisma statement. Ann. Intern. Med. 151, 264–269 (2009). 19622511 10.7326/0003-4819-151-4-200908180-00135

[bib29] Stroup, D. F. et al. Meta-analysis of observational studies in epidemiology: a proposal for reporting. JAMA 283, 2008–2012 (2000). 10789670 10.1001/jama.283.15.2008

[bib30] Duijts, L. Fetal and infant origins of asthma. Eur. J. Epidemiol. 27, 5–14 (2012). 22350146 10.1007/s10654-012-9657-y PMC3292726

