# Supplementary material for: Acid-suppressive medications during pregnancy and risk of asthma and allergy in the offspring: protocol for a systematic review
Source: NPJ Prim Care Respir Med. 2016 Mar 3;26:16001–. doi: 10.1038/npjpcrm.2016.1 (PMC4776038; doi:10.1038/npjpcrm.2016.1)
Supplement: Supplementary Appendix 1 [file npjpcrm20161-s1.doc]

# Appendix 1: Search strategy for Medline (Ovid)

1. exp Proton Pump Inhibitors/ OR *Gastroesophageal Reflux/ OR exp Anti-Ulcer Agents/ OR exp Histamine H2 Antagonists/ OR *Helicobacter Infections/

2. exp Antacids/ OR antacid.mp.

3. exp Aluminum Hydroxide/ae, tu [Adverse Effects, Therapeutic Use]

4. magnesium carbonate.mp.

5. magnesium trisilicate.mp.

6. hydrotalcite.mp.

7. Alginates.mp. OR exp Alginates/

8. Omeprazole/

9. Lansoprazole/

10. Esomeprazole/

11. Rabeprazole/

12. pantoprazole.mp.

13. Cimetidine/

14. Famotidine/

15. Nizatidine/

16. Ranitidine/

17. OR/ 1-16

18. exp pregnancy trimesters/

19. pregnancy/

20. antenatal.mp

21. Pregnancy Trimester, Third/ or exp Pregnancy/ or Pregnancy Trimester, First/ or Pregnancy Trimester, Second/ or Pregnancy Trimesters/ or pregnancy.mp.

22. exp Asthma/

23. asthma.mp.

24. wheez*.mp.

25. exp Bronchial Hyperreactivity/

26. airway hyperreactivity.mp.

27. bronchial disorder.mp.

28. lung function.mp.

29. respiratory function.mp.

30. ventilatory function.mp.

31. airway function.mp.

32. Vital Capacity/

33. Forced Expiratory Volume/

34. Peak Expiratory Flow Rate/

35. peak expiratory flow.mp.

36. exp hypersensitivity/

37. exp dermatitis, allergic contact/

38. exp hypersensitivity, immediate/

39. anaphylaxis/

40. conjunctivitis, allergic/

41. dermatitis, atopic/

42. exp food hypersensitivity/

43. exp respiratory hypersensitivity/

44. exp rhinitis, allergic/

45. exp urticaria/

46. angioedema/

47. eczema/

48. allergy.mp.

49. atopy.mp.

50. OR/ 22-49

51. Limit 50 to “all child (0-18 years)”

52. OR/ 18-21

53. 17 AND 51 AND
